# Production of oxygen free radicals by Ehrlich ascites tumour cells: effect of lipids

**DOI:** 10.1155/S0962935193000079

**Published:** 1993

**Authors:** Gopal K. Marathe, Cletus J. M. D'Souza

**Affiliations:** Department of Studies in Biochemistry Manasagangotri University of Mysore Mysore 570 006 India

## Abstract

Phorbol-12-myristate-13-acetate (PMA), calcium ionophore A23187 and platelet activating factor (PAF) stimulated the generation of oxygen free radicals (nitro-blue tetrazolium reduction) in Ehrlich ascites tumour (EAT) cells. PAF was effective at an optimal concentration of 4 μM, but was inhibited by BN 52021, a specific PAF antagonist. Lyso-PAF was ineffective. Inclusion of different lipids during incubation prior to the addition of PAF, resulted in the activation/inhibition of free radical generation. Among the phospholipids at a concentration of 50 μg/ml, the order of activation was phosphatidylserine > phosphatidylglycerol > phosphoinositides > phosphatidylinositol > phosphatidylethanolamine. Phosphatidylcholine was not effective, while sphingolipids were inhibitory. In addition, Ehrlich ascites tumour cells grown in mice under marginal vitamin A deficiency, showed an augmented production of free radicals compared to control cells. This was suppressed by exogenous addition of vitamin A or superoxide dismutase. These results suggest that membrane lipids and dietary factors like vitamin A probably function as physiological modulators in regulating the free radical generation.


Mediators of Inflammation, 2, 53–57 (1993)

### Production of oxygen free radicals by Ehrlich ascites tumour cells: effect of lipids

Gopal K. Marathe[CA] and
Cletus J. M. D'Souza

Department of Studies in Biochemistry, Manasagangotri, University of Mysore, Mysore–570 006, India

[CA] Corresponding Author

**PHORBOL-12-myristate-13-acetate (PMA), calcium ionophore A23187 and platelet activating factor (PAF) stimulated the generation of oxygen free radicals (nitro-blue tetrazolium reduction) in Ehrlich ascites tumour (EAT) cells. PAF was effective at an optimal concentration of 4 μM, but was inhibited by BN 52021, a specific PAF antagonist. Lyso-PAF was ineffective. Inclusion of different lipids during incubation prior to the addition of PAF, resulted in the activation/inhibition of free radical generation. Among the phospholipids at a concentration of 50 μg/ml, the order of activation was phosphatidylserine > phosphatidylglycerol > phosphoinositides > phosphatidylinositol > phosphatidylethanolamine. Phosphatidylcholine was not effective, while sphingolipids were inhibitory. In addition, Ehrlich ascites tumour cells grown in mice under marginal vitamin A deficiency, showed an augmented production of free radicals compared to control cells. This was suppressed by exogenous addition of vitamin A or superoxide dismutase. These results suggest that membrane lipids and dietary factors like vitamin A probably function as physiological modulators in regulating the free radical generation.**

**Key words:** Ehrlich ascites tumour cells, Oxygen free radicals, PAF, Vitamin A

### Introduction

Phagocytic cells kill invading microorganisms in a metabolic event characterized by a marked increase in oxygen consumption termed 'respiratory burst'.[1] The membrane bound enzyme NADPH-oxidase catalyzes this reaction.[2] This enzyme is dormant in resting cells, but can be activated by a wide variety of stimulants.[2,3] During the activation of NADPH-oxidase, oxygen free radicals (like the superoxide anion $O_2^-$) are generated.[1,2] Naturally occurring antioxidants like vitamin E, vitamin C and vitamin A can inhibit free radical generation.[4,5] In addition to these vitamins, membrane lipids are also known to modulate free radical generation.[6] As vitamin A has been known to affect membrane integrity[7] and to impair immunity,[8] there may be a relationship between membrane lipids, vitamin A and free radicals.

In order to test this relationship, the free radical generation in Ehrlich ascites tumour cells (EAT cells) using stimulants like phorbol-12-myristate-13-acetate (PMA) calcium ionophore A23187, and PAF in cells grown in vitamin A deficient and vitamin A sufficient animals has been studied. The effect of various lipids and stimulants on free radical generation in this cell line is reported in this paper.

### Materials and Methods

*Chemicals:* Platelet activating factor (1-*O*-hexadecyl-2-acetyl-Sn-glycero-3-phosphocholine (PAF), calcium ionophore A23187, phorbol-12-myristate-13-acetate (PMA), phosphatidylserine (PS), phosphatidylethanolamine (PE), phosphatidylinositol (PI), phosphatidylcholine (PC), phosphatidylglycerol (PG), sphingomyelin (SM), sphingosine (SS), phosphoinositides (PIP), and superoxide dismutase (SOD) of bovine erythrocytes were obtained from Sigma Chemical Co., USA. BN 52021 was a generous gift from Dr P. Braquet (Institute Henri Beaufour, France). BN 52021, PMA, and calcium ionophore were dissolved in DMSO. PAF and Lyso-PAF were dissolved in 0.9% NaCl containing 2.5 mg/ml BSA and diluted with Tyrode–Ringer's buffer (pH 7.5) just before use. Nitro-blue tetrazolium (NBT) was from SR Laboratories, India. All other chemicals were of analytical grade and solvents were redistilled before use. The

© 1993 Rapid Communications of Oxford Ltd concentration of DMSO never exceeded 0.1% v/v in these experiments. Such a concentration of DMSO did not interfere with the experiments.

*Development of vitamin A deficiency in mice and culture of EAT cells:* Weanling Swiss albino mice were made vitamin A deficient as reported earlier.[9] The animals were divided into three groups: group 1, vitamin A deficient; group 2, pair-fed controls; and group 3, controls receiving a normal commercial diet (supplied by Lipton, India Ltd). The pair-fed controls received the same diet as the vitamin A deficient mice, except 120 I.U. of retinyl palmitate was given orally three times a week. EAT cells were cultured in the peritoneal cavity of such mice (12–14 weeks old) by serial transplantation. Viability of the cells was determined by trypan blue dye exclusion and was greater than 95%.

*Determination of oxygen free radicals by NBT reduction* The release of oxygen free radicals was determined spectrophotometrically by measuring the reduction of NBT at 540 nm.[10] Freshly harvested EAT cells ($4 \times 10^6$ cells) were suspended in Tyrode–Ringer's buffer (pH 7.5) in a total volume of 1 ml, containing 0.25% BSA. The cells, untreated or stimulated by various stimulants, were incubated with NBT (60 nmol) for 20 min. The cells were washed with the same buffer and lysed by the addition of 2 ml of 1,4-dioxane and maintained in a boiling water bath for 8 min. The lysate was centrifuged at $250 \times g$ for 5 min and the extracted blue colour was read at 540 nm. A calibration curve of absorbance at 540 nm was obtained using PAF (4 μM), PMA (2 μM) and calcium ionophore (2 μM) as stimulants, and different concentrations of NBT (0–80 nmol) on EAT cells ($4 \times 10^6$).

*Addition of lipids:* The various lipids in chloroform dried under nitrogen were prepared at two concentrations, 12.5 μg/ml and 50 μg/ml, and sonicated. These lipids were added to cells ($4 \times 10^6$ per ml) in Tyrode–Ringer buffer (pH 7.5) containing 0.25% BSA. The EAT cells suspended in this buffer were pre-incubated with these lipids (PS, PE, PI, PC, PG, SM, SS and PIP) prior to the addition of PAF (4 μM). The neutral lipid (NL) glycolipid (GL) and phospholipid fractions (PL) from total lipid extract of EAT cells were separated as described in an earlier paper[11] and the effect of these lipids on free radical generation was also studied. The free radicals were also measured in the presence of vitamin A and SOD.

*Statistical analysis:* The results are expressed as means ± S.D. and significance was assessed using Student's *t* test.

### Results

*Production of vitamin A deficiency in mice:* Although the literature with respect to vitamin A deficiency in rat is extensive, reports on vitamin A deficiency in mice are scanty.[12] In fact, it was difficult to develop vitamin A deficient mice. Even though we were able to develop severe vitamin A deficiency in mice, the animals could not survive when EAT cells were injected intraperitoneally. Hence, a marginal vitamin A deficient condition was chosen in this study. It took 10–12 weeks to develop such a deficiency.

*Stimulation of free radicals in EAT cells by PAF and its inhibition by BN 52021:* A dose response curve showing the effect of increasing PAF concentrations on free radical generation in EAT cells grown in vitamin A deficient, pair-fed controls and in the control mice receiving the commercial diet is shown in Fig. 1. Although the optimal level of PAF required to stimulate the free radical generation is 4 μM in all three groups, the basal level of free radicals is more in vitamin A deficient mice (10.5 ± 10.8 per $10^6$ cells) compared with control cells (5.85 ± 1.50 per $10^6$ cells). The production of free radicals was a maximum at 20 min and then gradually declined (Fig. 2). The specific PAF antagonist BN 52021[13] progressively inhibited the free radical generation over the concentration range of 5–50 μM (Fig. 3).

*Effect of lipids on free radical generation:* The effect of various lipids on the generation of free radicals in EAT cells is shown in Fig. 4. When PE, PS, PI, PG and PIP were used in the incubation medium prior to the addition of PAF (4 μM), there was a

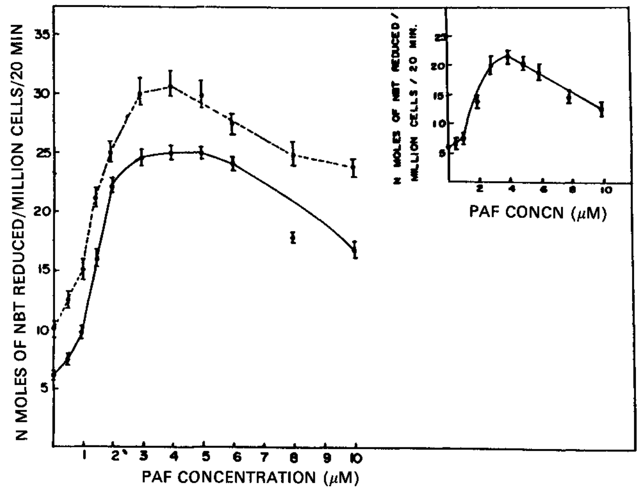

FIG. 1. Dose-response curves of the effect of increasing concentrations of PAF on the production of free radicals of EAT cells, grown under different conditions (---- vitamin A deficient and —— vitamin A sufficient). EAT cells were stimulated with the indicated concentrations of PAF (see Methods). Data are means ±S.D. of triplicated determinations. Inset: Effect of PAF on the production of free radicals by EAT cells grown in mice receiving a normal commercial laboratory diet (supplied by Lipton, India Ltd).

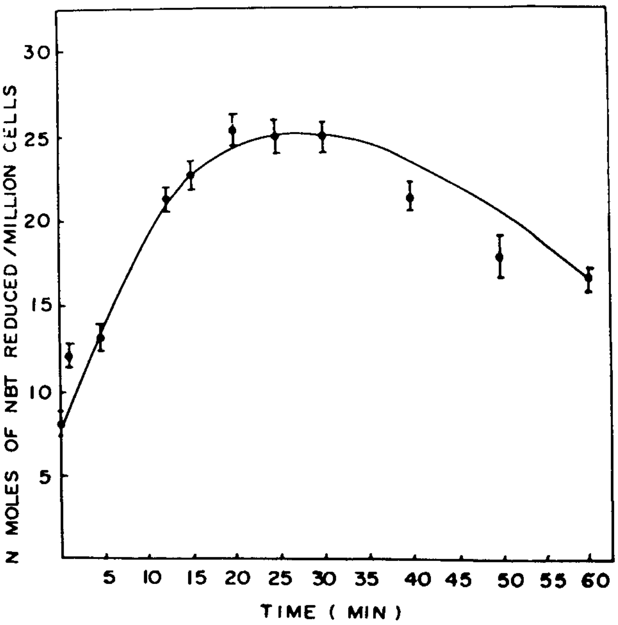

FIG. 2. Time-dependent formation of free radicals by EAT cells (grown in mice receiving a commercial diet). EAT cells ($4 \times 10^6$ cells) were stimulated with PAF (4 μM) for the indicated time intervals. Values are mean ±S.D. ($n = 3$).

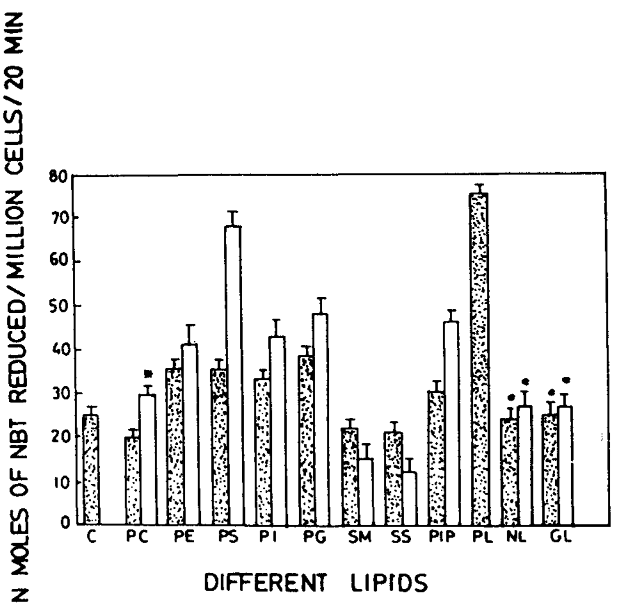

FIG. 4. Effect of lipids on free radical generation by EAT cells (grown in mice receiving a commercial diet) treated with PAF. Each lipid is included during pre-incubation with EAT ($4 \times 10^6$) cells at a concentration of either 12.5 μg/ml (shaded bars) or 50 μg/ml (open bars). The pre-incubation is followed by stimulation with PAF (4 μM) for 20 min (see Methods). C refers to control (no lipid is added). The values are mean ±S.D. ($n = 4$). *$p > 0.09$. ˙$p > 0.05$. Other values are significant ($p < 0.001$).

stimulation of free radical generation. PS was the most effective of the phospholipids used ($p < 0.001$). However, PC at the lower concentration (12.5 μg/ml) was inhibitory ($p < 0.001$), while at a higher concentration (50 μg/ml) the stimulatory effect was not significant ($p > 0.09$). Sphingolipids were inhibitory at both the concentrations used ($p < 0.001$). A more pronounced effect was seen when the phospholipid fraction (PL) derived from a total lipid extract of EAT cells was used. The free radical generation was increased 2.8 fold at a concentration of 12.5 μg/ml ($p < 0.001$). On the other hand, glycolipid (GL) and neutral lipid (NL) fractions derived from EAT cells were ineffective in free radical generation in PAF stimulated cells ($p > 0.05$). The effect of these lipids on unstimulated cells was examined, but they did not affect the basal level of free radicals (data not shown).

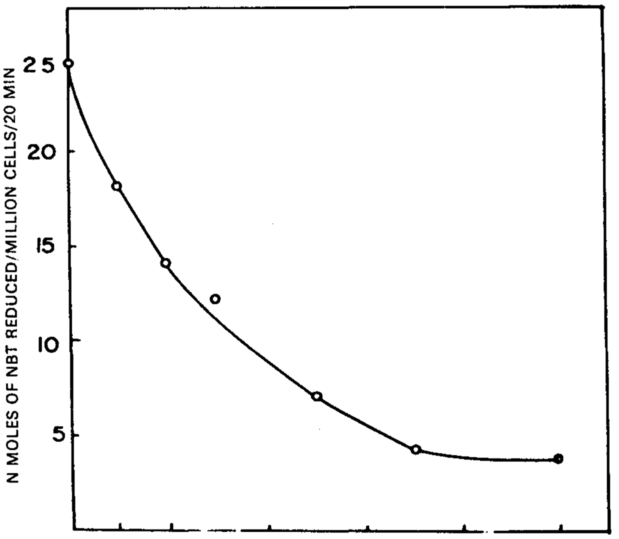

FIG. 3. Dose-response curve of the effect of increasing concentrations of BN 52021 on the production of free radicals by EAT cells (grown in mice receiving a commercial diet). $4 \times 10^6$ cells were pre-incubated with indicated concentrations of the antagonist for 10 min at 37°C and then stimulated with 4 μM PAF for 20 min at 37°C. Data are means of duplicate determinations from two independent experiments.

*Comparison of the free radical generation by different stimulants:* The effect of various stimulants on free radical generation is shown in Table 1. PMA appears to be the most effective activator of the respiratory burst, while PAF was the least. Exogenous addition of vitamin A and SOD suppress the enhanced respiratory burst, and also affect the basal levels of free radicals. However, lyso-PAF (the biologically inactive metabolite of PAF) totally failed to cause respiratory burst at both the concentrations used.

### Discussion

PAF has been reported to activate the respiratory burst in various cells such as macrophages, neutrophils and eosinophils.[14] In contrast, certain investigators could not detect the effect of PAF on free radical generation in human monocytes and rat alveolar macrophages.[14] Thus, some uncertainty still persists as to the role of PAF in free radical

**Table 1.** Effect of different stimulants on the production of free radicals in EAT cells grown in mice receiving a commercial diet under various conditions

| Stimulus | | Treatment | nmoles of NBT reduced/10$^6$ cells/20 min |
|---|---|---|---|
| None | | — | 6.25 ± 1.20 |
| PAF (4 μM) | | — | 22.0 ± 2.50 |
| Calcium ionophore A21387 | | | |
| | (2 μM) | — | 42.3 ± 3.20 |
| | (6 μM) | — | 51.0 ± 7.45 |
| PMA | (1 μM) | — | 36.4 ± 3.20 |
| | (2 μM) | — | 74.1 ± 2.58 |
| | (4 μM) | — | 85.0 ± 3.40 |
| Lyso-PAF | (5 μM) | — | 6.45 ± 1.35 |
| | (10 μM) | — | 6.95 ± 1.20 |
| None | | Vitamin A (10 μg/10$^6$ cells) | 4.20 ± 0.50 |
| | | Vitamin A (20 μg/10$^6$ cells) | 3.31 ± 1.12 |
| PAF | (4 μM) | Vitamin A (20 μg/10$^6$ cells) | 9.82 ± 5.20 |
| None | | SOD (5 units) | 3.6 ± 1.05 |
| PAF | (4 μM) | SOD (5 units) | 10.4 ± 1.18 |

Values are mean ± S.D. ($n = 3$).
(Whenever vitamin A and SOD are used, cells are pretreated with these and then stimulated.)

generation. It may not be a universal activator, but may act as an activator in a few cells. A new role for PAF has also been assigned in amplifying the respiratory burst induced by other stimulants.[15]

In a previous report[11] the authors showed that EAT cells produce PAF (95 pmol per 10$^6$ cells) on stimulation with calcium ionophore A23187 (10 μM). Here it is demonstrated that PAF produced by EAT cells can act on those cells and generate free radicals. Although the concentration of PAF used in the *in vitro* assay was far greater than PAF generated *in vivo* by an equivalent number of cells, it is possible that the local concentration of PAF may be even greater than the one employed in this study. Such an observation has been reported in rabbit leukocytes.[16] The production of free radicals is the main function of the phagocytic cells[1]; its generation in non-phagocytic cells such as human fibroblasts, and transformed cells such as human breast carcinoma[10] and EAT cells (present study), is interesting.

Lipids, especially the phospholipids and their metabolites, seem to play a crucial role in many cell functions, particularly in intracellular signalling.[17] Phospholipids also stimulate a variety of enzyme catalyzed oxidative reactions.[6] In the present study, most of the lipids used activate the respiratory burst oxidase except for sphingolipids which are inhibitory. Such an activation of the respiratory burst oxidase by PS was also observed by Tamura *et al.*[6]

Protein kinase C has been implicated as essential in activation of NADPH oxidase.[18] This is further supported by the fact that sphingolipids which are inhibitors of protein kinase C also inhibit NADPH oxidase.[18] However, Tamura *et al.*[6] have questioned the involvement of protein kinase C, since activation observed during PS addition could not be inhibited by EGTA, which is known to inhibit protein kinase C. Hence, it appears that although direct stimulation of NADPH oxidase by PS and its inhibition by sphingolipids is possible, involvement of protein kinase C cannot be ruled out.

Augmented production of free radicals during vitamin A deficiency is interesting. Vitamin A deficiency is known to alter the membrane integrity and to bring about associated changes[8,9] including changes in membrane lipid composition.[19] These effects may activate the respiratory burst oxidase. In fact, the basal level of free radicals is more during vitamin A deficiency and could be suppressed by exogenous addition of vitamin A, suggesting a role for vitamin A in free radical generation.

Besides PAF, other stimulants like PMA and calcium ionophore were also capable of eliciting the respiratory burst (Table 1). Although PAF appears to be a weak stimulant, it is a physiological one.

Activation/inhibition of the respiratory burst oxidase displayed by various phospholipids and dietary factors such as vitamin A probably play a regulatory role in free radical generation. As the respiratory burst is lethal for both the invader and the host, it should not be turned on unnecessarily. Membrane lipids and vitamin A probably regulate this event.

### References

1. Baggioli M, Wymann MP. Turning on the respiratory burst. *Trends Biochem Sci* 1990; **15**: 69–72.

2. Gabig TG, Babior BM. The $O_2$ forming oxidase responsible for the respiratory burst in human neutrophils. *J Biol Chem* 1979; **254:** 9070–9074.
3. Wymann MP, von Tscharner V, Deranleau DA, Baggiolini M. The onset of the respiratory burst in human neutrophils. *J Biol Chem* 1987; **262:** 12048–12053.
4. Katha VNR, Krishnamurthy S. Antioxidant function of vitamin A. *Int J Vit Nutr Res* 1977; **47:** 394–401.
5. Logani MK, Davis RE. Lipid oxidation: biological effects and antioxidants—A review. *Lipids* 1989; **15:** 485–495.
6. Tamura M, Tamura T, Tyagi SR, Lambeth JD. The superoxidase-generating respiratory burst oxidase of human neutrophil plasma membrane. *J Biol Chem* 1988; **263:** 17621–17626.
7. Anderson OR, Roels OA, Pfister RM. Dietary retinol and alpha-tocopherol and erythrocyte structure in rats. *Nature* 1967; **213:** 47–49.
8. Smith SM, Levy NS, Hayes CE. Impaired immunity in vitamin A-deficient mice. *J Nutr* 1987; **117:** 857–865.
9. Krause RF, Beamer KC, Lawrence C. Vitamin A deficiency and phospholipid metabolism. *Am J Clin Nutr* 1969; **22:** 27–32.
10. Das UN, Begin ME, Ellis G, Huang YS, Horrobin DF. Polyunsaturated fatty acids augment free radical generation in tumor cells *in vitro*. *Biochem Biophys Res Commun* 1987; **145:** 15–24.
11. Marathe GK, Krishnakantha TP, D'Souza CJM. PAF-acether (PAF) in Ehrlich ascites tumour cells. *J Lipid Med*, 1990: **2:** 257–262.
12. McCarthy PT, Cerecedo LR. Vitamin A deficiency in the mouse. *J Nutr* 1952; **46:** 361–376.
13. Braquet P, Spinnewyn B, Braquet M, Bourgain RH, Taylor JE, Etinne A, Drieu K. BN-52021 and related compounds: a new series of highly specific PAF-acether receptor antagonists isolated from *Ginkgo biloba*. *Blood Vessels* 1985; **16:** 559–572.
14. Rouis M, Nigon F, Chapman MJ. Platelet activating factor is a potent stimulant of the production of active oxygen species by human monocyte-derived macrophages. *Biochem Biophys Res Commun* 1988; **156:** 1293–1301.
15. Baggiolini M, Dewald B. Stimulus amplification of PAF and $LTB_4$ in human neutrophils. *Pharmacol Res Commun* 1986; **18:** 51–59.
16. Steward AG, Harris T. Platelet activating factor may participate in signal transduction process in rabbit leukocytes. *Lipids* 1991; **26:** 1044–1049.
17. Merill AH. Lipid modulators of cell function. *Nutrition Rev* 1989; **47:** 161–169.
18. Wilson E, Olcott MC, Bull RM, Merill AH, Lambeth JD. Inhibition of the oxidative burst in human neutrophils by sphingoid long chain bases. *J Biol Chem* 1986; **261:** 12616–12621.
19. Krause RF, Beamer KC, Plow JH. Phospholipid metabolism in vitamin A deficient rats. *J Nutr* 1971; **101:** 161–168.

ACKNOWLEDGEMENT. We thank Dr T. P. Krishnakantha (CFTRI-India) for his keen interest on vitamin A deficiency studies. GKM thanks University Grants Commission for a Senior Research Fellowship. CDS thanks CSIR, India, for a research grant.

**Received 24 August 1992; accepted in revised form 4 December 1992**

## References

[PDF_00434] Baggiolini M., Dewald B. (1986). Stimulus amplification by PAF and LTB4 in human neutrophils.. Pharmacol Res Commun.

[PDF_00398] Baggiolini M., Wymann M. P. (1990). Turning on the respiratory burst.. Trends Biochem Sci.

[PDF_00419] Das U. N., Begin M. E., Ells G., Huang Y. S., Horrobin D. F. (1987). Polyunsaturated fatty acids augment free radical generation in tumor cells in vitro.. Biochem Biophys Res Commun.

[PDF_00401] Gabig T. G., Babior B. M. (1979). The O2(-) -forming oxidase responsible for the respiratory burst in human neutrophils. Properties of the solubilized enzyme.. J Biol Chem.

[PDF_00406] Kartha V. N., Krishnamurthy S. (1977). Antioxidant function of vitamin A.. Int J Vitam Nutr Res.

[PDF_00417] Krause R. F., Beamer K. C., Lawrence C. (1969). Vitamin A deficiency and phospholipid metabolism.. Am J Clin Nutr.

[PDF_00443] Krause R. F., Beamer K. C., Plow J. H. (1971). Phospholipid metabolism in vitamin A-deficientrats.. J Nutr.

[PDF_00408] Logani M. K., Davies R. E. (1980). Lipid oxidation: biologic effects and antioxidants--a review.. Lipids.

[PDF_00422] Marathe G. K., Krishnakanta T. P., D'Souza C. J. (1990). PAF-acether (PAF) in Ehrlich ascites tumour cells.. J Lipid Mediat.

[PDF_00424] McCARTHY P. T., CERECEDO L. R. (1952). Vitamin A deficiency in the mouse.. J Nutr.

[PDF_00430] Rouis M., Nigon F., Chapman M. J. (1988). Platelet activating factor is a potent stimulant of the production of active oxygen species by human monocyte-derived macrophages.. Biochem Biophys Res Commun.

[PDF_00415] Smith S. M., Levy N. S., Hayes C. E. (1987). Impaired immunity in vitamin A-deficient mice.. J Nutr.

[PDF_00436] Stewart A. G., Harris T. (1991). Platelet-activating factor may participate in signal transduction processes in rabbit leukocytes.. Lipids.

[PDF_00410] Tamura M., Tamura T., Tyagi S. R., Lambeth J. D. (1988). The superoxide-generating respiratory burst oxidase of human neutrophil plasma membrane. Phosphatidylserine as an effector of the activated enzyme.. J Biol Chem.

[PDF_00440] Wilson E., Olcott M. C., Bell R. M., Merrill A. H., Lambeth J. D. (1986). Inhibition of the oxidative burst in human neutrophils by sphingoid long-chain bases. Role of protein kinase C in activation of the burst.. J Biol Chem.

[PDF_00403] Wymann M. P., von Tscharner V., Deranleau D. A., Baggiolini M. (1987). The onset of the respiratory burst in human neutrophils. Real-time studies of H2O2 formation reveal a rapid agonist-induced transduction process.. J Biol Chem.

